# Metabolic Surgery for Obese Type 2 Diabetes: Korean Multicenter Cohort Study

**DOI:** 10.1007/s11695-025-08374-7

**Published:** 2025-11-13

**Authors:** Young Suk Park, Soo Min Ahn, Sang Hyun Kim, Sung Il Choi, Kyung Won Seo, Han Hong Lee, Youngsung Suh, Ji Yeon Park, Sang Eok Lee, Sungsoo Park, Dong Jin Kim, In Cho, Yoo Min Kim, Songchang Shi, Tae Jung Oh, Yun-Suhk Suh, Ki Hyun Kim, Seungwan Ryu, Mi Kyung Kim, Do Joong Park, Seong-Ho Kong, Young Min Cho, In Gyu Kwon, Jong Suk Park, Minyoung Lee, Hyuk-Joon Lee

**Affiliations:** 1 Department of Surgery, Seoul National University Bundang Hospital, Seoul National University College of Medicine, Seongnam, Seoul, Korea, Republic of; 2 Department of Surgery, Gangnam Severance Hospital, Yonsei University College of Medicine, Seoul, Korea, Republic of; 3 Department of Surgery, Soonchunhyang University Hospital, Seoul, Korea, Republic of; 4 Department of Surgery, Kyung Hee University Hospital at Gangdong, Seoul, Korea, Republic of; 5 Department of Surgery, Kosin University College of Medicine, Busan, Korea, Republic of; 6 Department of Surgery, Seoul St. Mary’s Hospital, College of Medicine, The Catholic University of Korea, Seoul, Korea, Republic of; 7 Department of Family Medicine, Keimyung University School of Medicine, Daegu, Korea, Republic of; 8 Department of Surgery, School of Medicine, Kyungpook National University, Kyungpook National University Chilgok Hospital, Daegu, Korea, Republic of; 9 Department of General Surgery, Konyang University Hospital, Daejeon, Korea, Republic of; 10 Department of Surgery, Korea University College of Medicine, Seoul, Korea, Republic of; 11 Department of Gastrointestinal Surgery, Eunpyeong St. May’s Hospital, College of Medicine, The Catholic University of Korea, Seoul, Korea, Republic of; 12 Department of Surgery, Soonchunhyang University Bucheon Hospital, Soonchunhyang University College of Medicine, Bucheon, Korea, Republic of; 13 Department of Surgery, Republic of Korea Gastric Cancer Center, Yonsei Cancer Center, Yonsei University Health System, Yonsei University College of Medicine, Seoul, Korea, Republic of; 14 Department of Critical Care Medicine, Fujian Provincial Hospital, Fujian Provincial Hospital South Branch, Shengli Clinical Medical College of Fujian Medical University, Fuzhou University Affiliated Provincial Hospital, Fuzhou, China; 15 Department of Internal Medicine, Seoul National University College of Medicine and Seoul National University Bundang Hospital, Seongnam-si, Korea, Republic of; 16 Department of Surgery, Keimyung University School of Medicine, Daegu, Korea, Republic of; 17 Department of Internal Medicine, Keimyung University School of Medicine, Daegu, Korea, Republic of; 18 Department of Surgery and Cancer Research Institute, Seoul National University Hospital, Seoul National University College of Medicine, Seoul, Korea, Republic of; 19 Department of Internal Medicine, Seoul National University Hospital, Seoul National University College of Medicine, Seoul, Korea, Republic of; 20 Department of Internal Medicine, Gangnam Severance Hospital, Yonsei University College of Medicine, Seoul, Korea, Republic of; 21 Department of Internal Medicine, Yonsei University College of Medicine, Seoul, Korea, Republic of

**Keywords:** Sleeve gastrectomy, Roux-en-Y gastric bypass, Bariatric surgery, Diabetes mellitus, type 2, Propensity score

## Abstract

**Background:**

Metabolic/bariatric surgery is an effective treatment for obesity and type 2 diabetes mellitus (T2DM), with sleeve gastrectomy (SG) and Roux-en-Y gastric bypass (RYGB) being the most common procedures. However, comparative data on their metabolic benefits in East Asian populations are limited.

**Methods:**

This multicenter retrospective cohort study assessed outcomes of SG and RYGB in Korean adults (BMI ≥ 30 kg/m²) with T2DM who underwent surgery between January 2019 and June 2021. The primary outcome was complete T2DM remission at 1 and 2 years postoperatively. Follow-up data were available for 271 patients at 1 year and 169 at 2 years.

**Results:**

Unadjusted analysis showed lower complete remission rates in the RYGB group than the SG group at both 1 year (51.4% vs. 66.3%, *p* = 0.029) and 2 years (50.0% vs. 62.9%, *p* = 0.033). After adjustment using inverse probability of treatment weighting (IPTW), the pattern reversed in favor of RYGB (1 year: 67.5% vs. 48.2%; 2 years: 64.4% vs. 46.9%), though differences were not statistically significant (*p* = 0.127 and *p* = 0.373). Multivariable logistic regression identified shorter duration of T2DM and absence of insulin use as independent predictors of remission; surgical procedure type was not.

**Conclusions:**

After adjusting for baseline differences, no statistically significant difference in T2DM remission was observed between SG and RYGB at 1 and 2 years in Korean patients with BMI ≥ 30 kg/m². These findings should be interpreted with caution given the modest sample size and limited follow-up. Prospective studies are needed to validate these findings and support clinical decision-making.

**Supplementary Information:**

The online version contains supplementary material available at 10.1007/s11695-025-08374-7.

## Highlights

- Baseline-adjusted analysis revealed no statistically significant difference in diabetes remission between RYGB and SG at 1 and 2 years.
- Baseline diabetes severity significantly influenced remission outcomes, with longer diabetes duration and insulin use predicting lower remission rates.
- RYGB demonstrated greater total weight loss than SG, although this difference was not statistically significant at 2 years.
- These findings highlight the need for stratified randomized controlled trials to guide personalized metabolic surgery decisions in East Asian populations.

**Supplementary Information:**

The online version contains supplementary material available at 10.1007/s11695-025-08374-7.

## Introduction

 Obesity and type 2 diabetes mellitus (T2DM) are two of the most pressing public health challenges worldwide. Over the past four decades, the global prevalence of obesity has risen drastically, with more than 650 million adults classified as obese in 2016, according to the World Health Organization [1]. Alongside this increase, the number of people with T2DM has also grown rapidly, nearly tripling between 1980 and 2014, affecting more than 422 million individuals globally [2]. In addition to the significant individual burden, T2DM and obesity together constitute the concept of “diabesity,” reflecting the close interrelation between the two conditions [3]. In East Asia, including Korea, the prevalence of T2DM is also on the rise, with a growing need for effective therapeutic interventions [4].

Metabolic/bariatric surgery (MBS) is now recognized as one of the most effective treatments for both obesity and T2DM, offering substantial weight loss and metabolic improvements [5]. Current international guidelines recommend metabolic surgery as a treatment option for T2DM in patients with a body mass index (BMI) of 30 kg/m² or higher, especially in Asian populations where lower BMI thresholds are used to define obesity-related comorbidities [6, 7].

Multiple mechanisms have been proposed to explain the T2DM remission observed after bariatric surgery [8]. A key pathway involves increased secretion of glucagon-like peptide-1 (GLP-1), which boosts insulin secretion and suppresses glucagon. Additionally, surgery-induced changes in gut microbiota and bile acid metabolism may enhance insulin sensitivity and metabolic homeostasis. These effects highlight the role of MBS as a potent metabolic therapy beyond weight reduction.

Among MBS procedures, sleeve gastrectomy (SG) and Roux-en-Y gastric bypass (RYGB) are the most widely performed [9]. SG is technically simpler and associated with fewer complications [10], whereas RYGB generally offers greater weight loss and better glycemic outcomes [11, 12]. Given these distinct profiles, the choice of surgical procedure for a patient with T2DM and obesity remains a topic of debate. Surgeons must weigh the risks and benefits of each procedure to select the optimal approach for individual patients. However, randomized controlled trials have reported conflicting results regarding procedure choice for diabesity. The SLEEVEPASS and SM-BOSS trials suggested no significant difference in long-term diabetes remission between RYGB and SG [13, 14], whereas the Oseberg trial demonstrated superior remission outcomes with RYGB [15]. These discrepancies underscore the ongoing debate and highlight the need for population-specific evidence in East Asian patients.

Furthermore, most studies focus on Western populations, with limited evidence on the Asian population who have unique genetic and phenotypic characteristics influencing obesity and T2DM outcomes [16]. As East Asian populations tend to develop T2DM at lower BMI levels than their Western counterparts, the applicability of existing evidence to Asian patients may be limited [17, 18].

This study sought to compare the effects of SG and RYGB on glucose control, weight loss, and the incidence of postoperative complications in Korean patients with obesity and T2DM. Therefore, we aimed to provide robust, population-specific evidence that can guide surgical decision-making for patients with combined T2DM and obesity in East Asia.

## Methods

### Study Design and Population

This was a retrospective, multicenter cohort study including adults (≥ 18 years) who underwent sleeve gastrectomy (SG) or Roux-en-Y gastric bypass (RYGB) between January 2019 and June 2021. Eligible patients had a body mass index (BMI) ≥ 30.0 kg/m² and a diagnosis of type 2 diabetes mellitus (T2DM) at the time of surgery. Patients with prior bariatric surgery or abdominal operations potentially affecting outcomes were excluded. In Korea, where the incidence of gastric cancer is relatively high, primary resectional gastric bypass is occasionally performed due to concerns about remnant stomach malignancy [19]. As clinical outcomes between resectional gastric bypass and conventional RYGB are comparable, both procedures were included under the RYGB category [20, 21]. All included operations were performed by board-certified metabolic and bariatric surgeons accredited by the Korean Society for Metabolic and Bariatric Surgery (KSMBS). We summarized center-level enrollment (number of patients per center and by procedure) and report these counts in Supplementary Table 1. The study was approved by the Institutional Review Board (IRB) [blinded] and by IRBs of all participating centers. Informed consent was waived due to the retrospective design and use of de-identified data.

**Table 1:** Baseline characteristics

	Sleeve gastrectomy (N=292)	Roux-en-Y Gastric bypass (N=143)	*p*
Sex, female (%)	179 (61.3)	99 (69.2)	0.172
Age, yrs	41.7 ± 12.1	45.3 ± 12.2	0.003
Smoking (%)			0.002
Never-smoker	196 (67.1)	96 (67.1)	
Current smoker	66 (22.6)	18 (12.6)	
Ex-smoker	30 (10.3)	29 (20.3)	
Operation time, min	118.4 ± 40.2	165.7 ± 64.6	< 0.001
Hospital stay, days	5.7 ± 3.5	6.2 ± 2.6	0.093
Postoperative stay, days	4.3 ± 2.9	4.8 ± 2.2	0.079
Height, cm	166.5 ± 9.6	164.9 ± 8.8	0.076
Weight, kg	112.6 ± 24.5	103.9 ± 20.2	< 0.001
BMI, kg/m^2^	40.3 ± 6.7	38.0 ± 5.5	< 0.001
DM duration, yrs	3.1 ± 4.7	6.1 ± 7.1	< 0.001
Insulin use (%)	21 (7.2)	48 (33.6)	< 0.001
Insulin: Total daily dose, UNIT	64.5 ± 71.6	49.3 ± 33.9	0.392
Chronic heart disease (%)	26 (8.9)	23 (16.1)	0.039
Stroke (%)	5 (1.7)	3 (2.1)	0.999
Peripheral arterial disease (%)	12 (4.1)	9 (6.3)	0.447
Retinopathy (%)			0.002
Yes	9 (3.1)	13 (9.1)	
Unknown	65 (22.3)	44 (30.8)	
Nephropathy (%)			< 0.001
Yes	9 (3.1%)	20 (14.0%)	
Unknown	55 (18.8%)	30 (21.0%)	
Neuropathy (%)			0.038
Yes	10 (3.4%)	13 (9.1%)	
Unknown	41 (14.0%)	22 (15.4%)	
Hypertension (%)	201 (68.8)	104 (72.7)	0.471
Systolic BP, mmHg	129.2 ± 16.3	133.9 ± 18.7	0.008
Diastolic BP, mmHg	77.5 ± 12.5	81.3 ± 12.4	0.003
Dyslipidemia (%)	177 (60.6)	109 (76.2)	0.002
NAFLD (%)	170 (58.2)	103 (72.0)	0.007
OSA (%)	122 (41.8)	82 (57.3)	0.003
Psychotic disease (%)	67 (22.9)	35 (24.5)	0.815
GERD, subjective (%)	80 (27.4)	38 (26.6)	0.947
Lung disease (%)	15 (5.1)	4 (2.8)	0.383
Musculoskeletal disease (%)	60 (20.5)	23 (16.1)	0.326
Hiatal hernia (%)	23 (7.9)	8 (5.6)	0.514
C-peptide, ng/mL	5.0 ± 3.3	4.0 ± 2.7	0.001
Insulin, uIU/mL	32.6 ± 36.8	31.0 ± 47.1	0.734
HbA1C, %	7.2 ± 1.3	8.0 ± 1.5	< 0.001
Fasting blood glucose, mg/dL	169.3 ± 83.8	165.7 ± 61.1	0.662
PP2, mg/dL	228.5 ± 80.5	277.8 ± 90.8	0.004

*BMI* body mass index, *DM* diabetes mellitus, *NAFLD* non-alcoholic fatty liver disease, *OSA* obstructive sleep apnea, *PP2* postprandial plasma glucose at 2 hours

### Surgical Technique

SG was performed using a 36–38 Fr calibration bougie, with the gastric resection starting approximately 4–5 cm proximal to the pylorus to create a tubular stomach along the lesser curvature. RYGB was performed with a small gastric pouch of approximately 25–30 mL in volume, a gastrojejunostomy diameter of 2.5–3.0 cm created using a linear stapler, an alimentary limb length of 100–150 cm, and a biliopancreatic limb length of about 100 cm. In both procedures, concomitant hiatal hernia was repaired when present.

### Outcome Measures

The primary outcome was the rate of complete T2DM remission at 1 and 2 years postoperatively, defined as no diabetes medication use with HbA1c < 6.0% or FBG < 100 mg/dL. Secondary outcomes included partial remission and improvement of T2DM, remission or improvement of hypertension and dyslipidemia, BMI changes, and postoperative complications. All other definitions and complication classification followed the criteria established by the American Society of Metabolic and Bariatric Surgery [22].

### Data Collection and Statistical Analysis

Relevant variables were extracted from the KSMBS registry using a study-specific case report form. Follow-up data and other variables not available in the registry were newly obtained through trained staff data entry and verified through routine audits.

For bivariate analysis comparing baseline characteristics between SG and RYGB groups, chi-square or Fisher’s exact tests were used for categorical variables, and Student’s t-tests or Mann–Whitney U tests were applied for continuous variables, depending on the distribution of the data.

To adjust for baseline differences between SG and RYGB groups, inverse probability of treatment weighting (IPTW) was conducted using propensity scores from logistic regression. Covariates included sex, age, smoking, BMI, hypertension, dyslipidemia, diabetes duration, diabetic complications, insulin use, non-alcoholic fatty liver disease (NAFLD), sleep apnea, psychotic disorder, C-peptide, and HbA1c. IPTW was performed separately for 1-year and 2-year cohorts. We selected IPTW rather than propensity score matching because IPTW allows estimation of the average treatment effect in the entire study population and retains all eligible patients, thereby preserving statistical power in our modest sample size. Weighted linear regression and weighted chi-square tests were used to compare outcomes between groups.

To identify predictors of T2DM remission at 1 and 2 years, we fitted multivariable logistic regression models on the unweighted cohort. The outcome was diabetic remission—defined as achieving either complete or partial remission—to increase the number of events and ensure adequate statistical power. Covariates were age, BMI, diabetes duration, insulin use, C-peptide, aspartate aminotransferase, alanine aminotransferase, and procedure type.

As sensitivity analyses, we further accounted for the multicenter design in two ways: (1) by estimating cluster-robust standard errors with center as the clustering unit for IPTW-weighted outcome comparisons, and (2) by fitting mixed-effects logistic regression models with a random intercept for center for the remission outcomes. In addition, for the primary 1-year endpoint, we generated a center-stratified forest plot with odds ratios and 95% CIs, and assessed heterogeneity using Cochran’s Q and I²; a random-effects pooled estimate was also reported.

Statistical analyses were performed using R (R Foundation, Vienna, Austria).

## Results

**Fig. 1:**
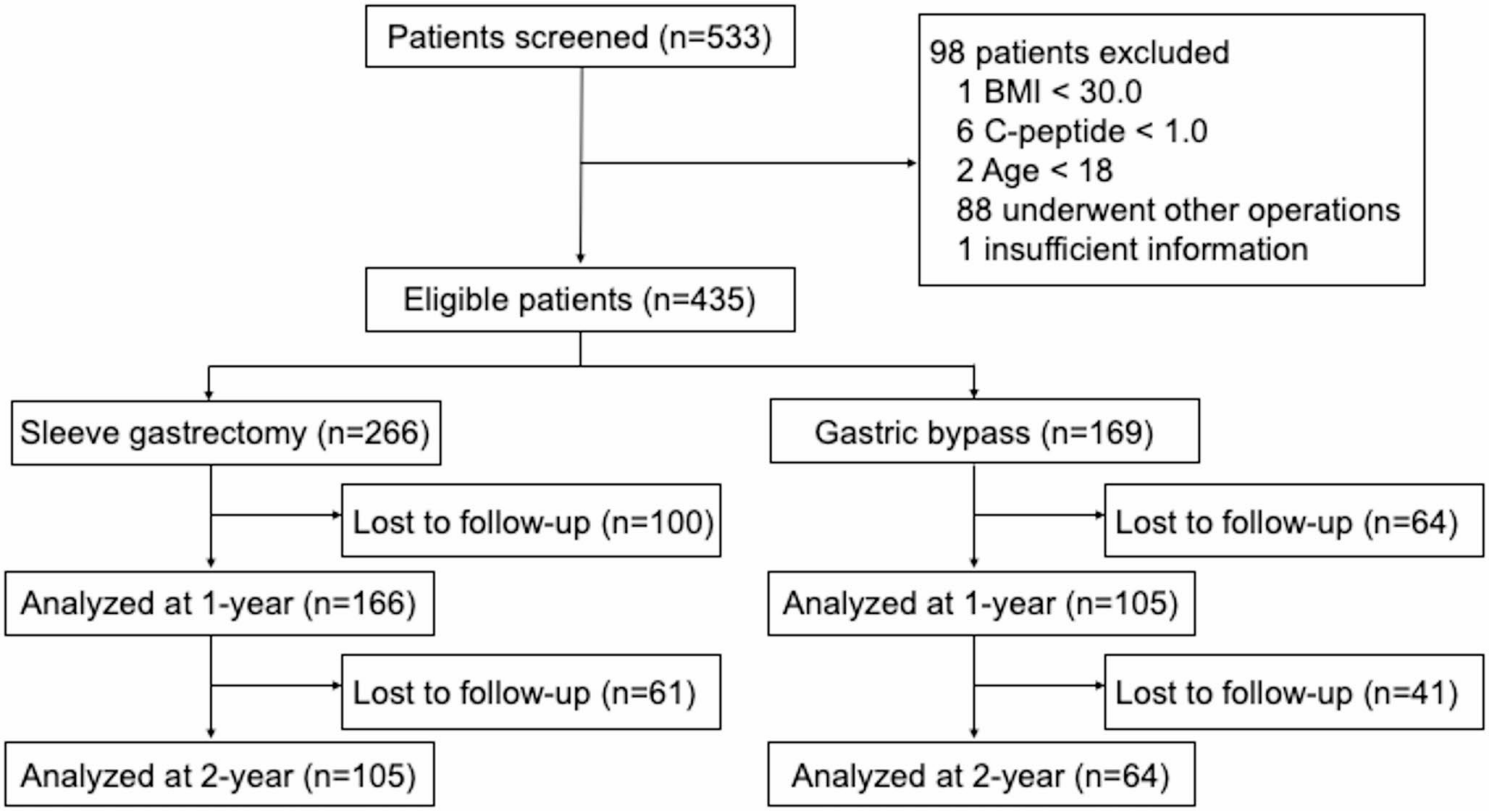
Flow diagram of patient selection and follow-up

A total of 435 eligible patients were included in the final analysis. Of these, 271 (166 SG and 105 RYGB) had 1-year follow-up data, and 169 (105 SG and 64 RYGB) were assessed at 2 years (Fig. 1). Patient enrollment across the 14 participating centers ranged from 5 to 94 individuals.

### Baseline Characteristics

The RYGB group was older (45.3 ± 12.2 vs. 41.7 ± 12.1 years, *p* = 0.003) and had lower BMI (38.0 ± 5.5 vs. 40.3 ± 6.7 kg/m², *p* < 0.001). Operation time was longer in RYGB (165.7 ± 64.6 vs. 118.4 ± 40.2 min, *p* < 0.001). T2DM was more severe in RYGB patients, with longer diabetes duration (6.1 ± 7.1 vs. 3.1 ± 4.7 years; median 4.0 years [IQR 1.0–9.0] vs. 1.0 years [IQR 0.1–3.0], *p* < 0.001) and higher insulin use (33.6% vs. 7.2%, *p* < 0.001). Diabetic complications were more frequent, including retinopathy (9.1% vs. 3.1%, *p* = 0.002), nephropathy (14.0% vs. 3.1%, *p* < 0.001), and neuropathy (9.1% vs. 3.4%, *p* = 0.038). HbA1c was higher (8.0 ± 1.5% vs. 7.2 ± 1.3%, *p* < 0.001), while C-peptide was lower (4.0 ± 2.7 vs. 5.0 ± 3.3 ng/mL, *p* = 0.001) (Table 1).

After applying IPTW using propensity scores, all baseline characteristics including BMI, diabetic duration, insulin use, and preoperative HbA1c showed no significant differences between groups for both 1-year and 2-year follow-up cohorts, indicating effective elimination of baseline differences (Supplementary Tables 2 and 3).

**Table 2:** Body weight loss and diabetes remission outcomes

	Before IPTW				After IPTW		
	Sleeve gastrectomy	Gastric bypass		*p*	Sleeve gastrectomy	Gastric bypass	*p*
Postoperative 1-year	(*N* = 166)	(*N* = 105)			(*N* = 308.4)	(*N* = 271.9)	
Body weight, kg	84.7 ± 18.1	77.0 ± 15.0		<0.001	82.6 ± 16.0	83.5 ± 20.4	0.860
BMI, kg/m^2^	30.7 ± 5.5	28.3 ± 3.8		<0.001	30.1 ± 4.8	29.9 ± 4.6	0.874
TWL, %	23.6 ± 9.0	24.6 ± 7.5		0.318	21.0 ± 9.5	26.1 ± 7.4	0.025
Diabetes remission				0.029			0.127
Complete remission	110 (66.3%)	54 (51.4%)			148.5(48.2%)	183.7(67.5%)	
Partial remission	15 (9.04%)	7 (6.67%)			22.1(7.2%)	16.9(6.2%)	
Improvement	37 (22.3%)	40 (38.1%)			130.8(42.4%)	66.5(24.5%)	
No change	4 (2.41%)	4 (3.81%)			7.0(2.3%)	4.8(1.8%)	
Postoperative 2-year	(*N* = 105)	(*N* = 64)			(*N* = 196.6)	(*N* = 145.8)	
Body weight, kg	85.0 ± 20.4	75.5 ± 15.1		0.001	81.6 ± 17.2	78.3 ± 14.6	0.307
BMI, kg/m^2^	30.7 ± 5.51	27.8 ± 3.70		<0.001	29.7 ± 4.78	28.4 ± 3.66	0.247
TWL, %	23.3 ± 9.34	25.4 ± 9.43		0.169	20.2 ± 9.21	24.5 ± 9.49	0.136
Diabetes remission				0.033			0.373
Complete remission	66 (62.9%)	32 (50.0%)			92.2 (46.9%)	93.9 (64.4%)	
Partial remission	12 (11.4%)	3 (4.7%)			13.8 (7.0%)	3.9 (2.7%)	
Improvement	25 (23.8%)	28 (43.8%)			88.0 (44.8%)	46.8 (32.1%)	
No change	1 (1.0%)	1 (1.6%)			1.5 (0.7%)	1.2 (0.8%)	
Aggravation	1 (1.0%)	0			1.1 (0.5%)	0	

*BMI* body mass index, *TWL* total weight loss

**Table 3:** Predictable factors for diabetes remission

A. Postoperative 1-year				
	Univariable OR (95% CI)	*p*	Multivariable-adjusted OR (95% CI)	*p*
Age (continuous)	0.97 (0.95–0.99)	0.002	0.99 (0.96–1.03)	0.717
Operative procedure				
Sleeve gastrectomy	1 [Reference]		1 [Reference]	
Roux-en-Y Gastric bypass	0.45 (0.27–0.77)	0.003	1.63 (0.74–3.79)	0.239
BMI (continuous)	1.07 (1.03–1.13)	0.003	1.05 (0.98–1.13)	0.150
DM duration (continuous)	0.74 (0.67–0.80)	< 0.001	0.76 (0.68–0.84)	< 0.001
Insulin use	0.09 (0.04–0.20)	< 0.001	0.29 (0.10–0.84)	0.023
C-peptide (continuous)	1.16 (1.04–1.33)	0.013	0.99 (0.88–1.13)	0.882
AST (continuous)	1.01 (1.00–1.02)	0.030	1.00 (0.98–1.02)	0.954
ALT (continuous)	1.01 (1.00–1.02)	0.006	1.00 (0.98–1.01)	0.856
B. Postoperative 2-year				
	Univariable OR (95% CI)	*p*	Multivariable-adjusted OR (95% CI)	*p*
Age (continuous)	0.94 (0.91–0.96)	< 0.001	0.98 (0.93–1.03)	0.438
Operative procedure				
Sleeve gastrectomy	1 [Reference]		1 [Reference]	
Roux-en-Y Gastric bypass	0.42 (0.21–0.80)	0.009	0.76 (0.29–2.04)	0.576
BMI (continuous)	1.13 (1.07–1.22)	< 0.001	1.13 (1.02–1.28)	0.044
DM duration (continuous)	0.81 (0.74–0.87)	< 0.001	0.86 (0.77–0.94)	0.002
Insulin use	0.09 (0.03–0.21)	< 0.001	0.25 (0.06–0.86)	0.033
C-peptide (continuous)	1.37 (1.15–1.71)	0.002	1.10 (0.91–1.37)	0.333
AST (continuous)	1.02 (1.01–1.04)	0.013	1.00 (0.97–1.04)	0.835
ALT (continuous)	1.02 (1.01–1.04)	0.001	1.01 (0.99–1.04)	0.495

*OR* odds ratio, *BMI* body mass index, *DM* diabetes mellitus, *AST* aspartate aminotransferase, *ALT* alanine aminotransferase

### Postoperative Morbidity and Mortality

Postoperative morbidity was similar between groups, with no significant differences in complication rates. Major early complications occurred in 7.0% of RYGB and 3.8% of SG patients (*p* = 0.216), while major late complications were reported in 1.4% and 0.7%, respectively (*p* = 0.843). Staple-line leakage occurred in 0.7% of SG patients and none in RYGB (*p* = 0.812), while bleeding was noted in 1.4% of RYGB and 1.7% of SG (*p* = 0.999); all cases represented intra-abdominal bleeding, with one SG patient requiring angiographic embolization and another requiring reoperation, while the remaining cases were managed conservatively with transfusion and supportive care. Prolonged hospital stay (more than 7 days) was more frequent in the RYGB group (7.0% vs. 2.7%), though not statistically significant (*p* = 0.066) (Supplementary Table 4).

**Table 4:** Improvement in metabolic comorbidities

	Postoperative 1-year outcomes			Postoperative 2-year outcomes		
	Sleeve gastrectomy (*N* = 166)	Roux-en-Y Gastric bypass (*N* = 105)	*p*	Sleeve gastrectomy (*N* = 105)	Roux-en-Y Gastric bypass (*N* = 64)	*p*
Hypertension						
Complete remission	20 (20.4%)	23 (30.7%)	0.156	16 (20.5%)	20 (31.7%)	0.359
Partial remission	30 (30.6%)	17 (22.7%)		26 (33.3%)	22 (34.9%)	
Improvement	38 (38.8%)	29 (38.7%)		26 (33.3%)	14 (22.2%)	
No change	10 (10.2%)	4 (5.3%)		8 (10.3%)	4 (6.3%)	
Progression	0	2 (2.7%)		2 (2.6%)	3 (4.8%)	
Dyslipidemia						
Remission	38 (33.3%)	39 (49.4%)	0.055	39 (38.2%)	31 (50.8%)	0.214
Improvement	62 (54.4%)	34 (43.0%)		31 (30.4%)	12 (19.7%)	
No change	6 (5.3%)	5 (6.3%)		30 (29.4%)	15 (24.6%)	
Progression	8 (7.0%)	1 (1.3%)		2 (2.0%)	3 (4.9%)	

Readmission (5.6% RYGB vs. 4.5% SG, *p* = 0.776) and reoperation rates (2.1% vs. 0.7%, *p* = 0.412) were also comparable. Reoperation causes included 2 incisional hernias in each group, with 1 additional bleeding case in the RYGB group. No mortality was reported in either group.

### Weight Loss Outcomes

Before IPTW adjustment, the RYGB group had significantly lower body weight and BMI at 1 year (77.0 ± 15.0 kg vs. 84.7 ± 18.1 kg, *p* < 0.001; BMI: 28.3 ± 3.8 vs. 30.7 ± 5.5 kg/m², *p* < 0.001) and 2 years (75.5 ± 15.1 kg vs. 85.0 ± 20.4 kg, *p* = 0.001; BMI: 27.8 ± 3.7 vs. 30.7 ± 5.5 kg/m², *p* < 0.001). However, %TWL was comparable at 1 year (24.6% vs. 23.6%, *p* = 0.318) and 2 years (25.4% vs. 23.3%, *p* = 0.169).

After IPTW adjustment, body weight and BMI were similar at 1 year (83.5 vs. 82.6 kg, *p* = 0.860; BMI: 29.9 vs. 30.1 kg/m², *p* = 0.874), but %TWL was significantly higher in the RYGB group (26.1% vs. 21.0%, *p* = 0.025). At 2 years, %TWL remained higher in RYGB (24.5% vs. 20.2%), though not statistically significant (*p* = 0.136) (Table 2).

#### Diabetes Remission

Before IPTW adjustment, the SG group had significantly higher complete remission rates at 1 year (66.3% vs. 51.4%, *p* = 0.029) and 2 years (62.9% vs. 50.0%, *p* = 0.033), while improvement was more frequently observed in the RYGB group (1-year: 38.1% vs. 22.3%; 2-year: 43.8% vs. 23.8%).

After IPTW adjustment, the diabetes remission distribution patterns were reversed but became statistically non-significant. At the 1-year follow-up, complete remission was more common in the RYGB group compared to the SG group (67.5% [*n* = 183.7] vs. 48.2% [*n* = 148.5], *p* = 0.127), and this trend remained at 2 years (64.4% [*n* = 93.9] vs. 46.9% [*n* = 92.2], *p* = 0.373) (Table 2).

When center-clustered robust standard errors were applied, p-values were further attenuated (e.g., 1-year complete remission *p* = 0.225; 2-year complete remission *p* = 0.489), confirming that remission outcomes did not differ significantly between groups after accounting for within-center correlation. The 1-year center-stratified forest plot further demonstrated consistency across centers, with a pooled random-effects OR of 0.36 (95% CI 0.15–0.84). Between-center heterogeneity was modest (Q = 13.2, *p* = 0.21; I² = 38.4%), indicating no significant inconsistency across centers (Supplementary Fig. 1).

### Multivariable Analysis of Diabetes Remission Predictors

At 1 year, shorter T2DM duration (odds ratio [OR]: 0.76; 95% confidence interval [CI]: 0.68–0.84; *p* < 0.001) and absence of insulin use (OR: 0.29; 95% CI: 0.10–0.84; *p* = 0.023) were significant predictors of diabetes remission. Surgical procedure type (RYGB vs. SG) was not significant (OR: 1.63; 95% CI: 0.74–3.79; *p* = 0.239) (Table 3 A).

At 2 years, shorter diabetes duration (OR: 0.86; 95% CI: 0.77–0.94; *p* = 0.002) and absence of insulin use (OR: 0.25; 95% CI: 0.06–0.86; *p* = 0.033) remained significant predictors of remission, while higher preoperative BMI (adjusted OR: 1.13, 95% CI: 1.02–1.28, *p* = 0.044) was also associated with greater likelihood of remission. The surgical procedure did not influence T2DM remission at 2 years postoperatively (OR 0.76, 95% CI 0.29–2.04, *p* = 0.576) (Table 3B).

In a sensitivity analysis using mixed-effects logistic regression with a random intercept for center, results were unchanged: procedure type remained non-significant at both 1 and 2 years, whereas diabetes duration and insulin use were consistently associated with remission. The association between BMI and 2-year remission was attenuated (*p* = 0.067) (Supplementary Table 5).

### Hypertension and Dyslipidemia Remission

Hypertension remission rates did not differ significantly between groups at 1 year or 2 years. At 1 year, complete remission was higher in the RYGB group (30.7% vs. 20.4%, *p* = 0.156), while partial remission and improvement were similar. A similar pattern was observed at 2 years (31.7% vs. 20.5%, *p* = 0.359).

For dyslipidemia, the RYGB group showed higher remission rates at both 1 year (49.4% vs. 33.3%, *p* = 0.055) and 2 years (50.8% vs. 38.2%, *p* = 0.214), though not statistically significant. Dyslipidemia progression was slightly more frequent in the SG group at 1 year (7.0% vs. 1.3%), while at 2 years the rates were similar (2.0% vs. 4.9%). (Table 4).

## Discussion

This study found that the type of surgical procedure was not significantly associated with T2DM remission at 1 or 2 years in Korean patients with BMI ≥ 30 kg/m². Even after IPTW adjustment, no significant difference in remission rates was observed. In multivariable analysis, diabetes duration and insulin use were significant predictors, while procedure type was not. These findings align with some prior studies but differ from others, highlighting ongoing debate over the metabolic superiority of RYGB vs. SG.

Several landmark randomized controlled trials (RCTs), including the SLEEVEPASS and SM-BOSS trials, have offered key insights into the metabolic benefits of RYGB and SG [13, 14]. Both the SLEEVEPASS and SM-BOSS trials primarily assessed long-term weight loss between procedures, with T2DM remission as a secondary outcome. At 5 years, neither study found a significant difference in remission rates, and the SLEEVEPASS trial confirmed this at 10 years [11]. However, as weight loss was the primary endpoint, these trials had limited power to detect differences in diabetes outcomes. Thus, the lack of significant differences in remission should not be viewed as definitive evidence of equal efficacy between procedures for T2DM.

The Oseberg trial, a RCT with T2DM remission as the primary endpoint, demonstrated that RYGB was significantly more effective than SG at both 1- and 3-year follow-ups [12, 15]. The result revealed stronger evidence to support the conclusion that RYGB may have superior benefits. A RCT in East Asia reported findings consistent with this trial, showing significantly higher remission rates with RYGB [23].

The findings of our study aligned with SLEEVEPASS and SM-BOSS trials showing no difference in diabetes remission but superior RYGB weight loss outcomes [13, 14]. However, our study had notable baseline imbalances between SG and RYGB groups prior to adjustment, particularly in T2DM severity parameters. Interestingly, patients in the RYGB group had significantly lower baseline BMI than those in the SG group, which may appear counterintuitive. This likely reflects the fact that all patients in this study underwent surgery primarily for the treatment of diabetes, rather than weight loss alone. In this real-world context, surgeons tended to preferentially select bypass procedures for patients with longer disease duration, greater severity, and higher rates of insulin use, even if their BMI was relatively lower. This practice pattern is consistent with prior reports, including a multinational Asian study demonstrating superior T2DM remission outcomes with bypass-type procedures compared to non-bypass procedures [24].

While IPTW and multivariable analyses mitigated confounding effects, residual confounding cannot be ruled out. Before adjustment, SG appeared to show higher complete T2DM remission rates; however, after IPTW, this finding reversed, with RYGB showing higher remission rates, although without statistical significance. This striking reversal directly illustrates the impact of baseline imbalances and underscores that the apparent procedure effect is strongly confounded by diabetes severity. Importantly, this shift itself is clinically meaningful, suggesting that if groups were better matched for baseline severity, a significant advantage of RYGB might have been observed. Therefore, the absence of a statistically significant difference should be interpreted with caution, acknowledging the retrospective design and the inherent challenges of fully accounting for disease severity.

Another factor explaining comparable diabetes remission rates between procedures may be SG’s intrinsic efficacy in improving glycemic control. Both procedures promote glycemic control through mechanisms like increased GLP-1 secretion, gut microbiota changes, and bile acid metabolism [25–27]. Thus, the differences in diabetes remission between RYGB and SG may exist but may not be large enough to reach statistical significance in a relatively small sample size study [28].

The evidence comparing RYGB and SG impact on T2DM remission remains complex and debated. A notable limitation of aforementioned studies is inadequate accounting for baseline T2DM severity. T2DM severity significantly influences remission likelihood after bariatric surgery—patients with mild T2DM achieve remission regardless of procedure, while severe diabetes patients may only improve or remain unchanged [29–31]. This highlights the necessity of stratifying patients by diabetic severity when evaluating comparative metabolic effectiveness of RYGB and SG. A study combining SLEEVEPASS and SM-BOSS data addressed this by categorizing patients using the Individualized Metabolic Surgery (IMS) score, finding no significant differences in remission rates between procedures even after severity stratification. However, authors acknowledged potential underpowering in smaller subgroups [32]. Therefore, prospective trials stratifying patients by diabetic severity are needed.

In line with these observations, we also performed supplementary analyses using the IMS score in our cohort. When stratified into mild, moderate, and severe diabetes groups, remission rates did not differ significantly between SG and RYGB within each stratum (Supplementary Table 6). Moreover, in a multivariable logistic regression including IMS score along with established covariates, IMS was independently associated with remission (OR 0.969, 95% CI 0.945–0.993, *p* = 0.011), whereas procedure type showed only a non-significant trend favoring RYGB (Supplementary Table 7). These findings provide additional support that baseline diabetes severity plays an important role in determining postoperative remission, and they suggest that incorporating severity stratification may be important in future studies.

This study has several limitations. First, its retrospective design makes it subject to inherent selection bias and residual confounding despite statistical adjustments. We attempted to mitigate confounding by applying IPTW with comprehensive covariates but the possibility of unmeasured confounding remains. Therefore, the findings should be interpreted with caution. In addition, covariate balance after weighting was not perfect. Balance was achieved (standardized mean differences < 0.1) for many variables including DM duration, C-peptide, and HbA1c in the 1-year cohort, but in the 2-year cohort the smaller sample size and larger baseline differences limited the ability of weighting to fully eliminate imbalance. Still, key variables such as BMI and C-peptide achieved good balance, supporting comparability for the main metabolic outcomes. Second, the sample size and follow-up duration were limited, which may have reduced the statistical power to detect modest but clinically meaningful differences. Thus, the non-significant findings after IPTW adjustment should be viewed in light of potential Type II error. Third, attrition was considerable at both 1 and 2 years, with follow-up rates of 62.3% (271/435) and 38.9% (169/435), respectively. This raises the possibility of attrition bias. However, a comparison of baseline characteristics between completers and non-completers showed no major differences other than a higher proportion of female patients at 1 year and shorter diabetes duration among those lost at 2 years (Supplementary Table 8), suggesting that attrition bias is unlikely to have substantially influenced the overall findings. Another limitation is the definition of complete remission. We used a stricter criterion (HbA1c < 6.0% and fasting glucose < 100 mg/dL without medications) based on the standardized outcomes reporting recommendations [22], whereas many studies adopt the ADA definition (HbA1c < 6.5% without therapy) [33]. This heterogeneity in remission definitions may reduce direct comparability with other reports. Furthermore, our study did not include an analysis of relapse, and the 2-year follow-up horizon is insufficient to determine the long-term durability of remission. Longer-term studies are needed to evaluate relapse and sustained glycemic outcomes after SG and RYGB. Lastly, the RYGB group included both conventional and resectional gastric bypass. In Korea, resectional gastric bypass is occasionally performed after patients are informed about the risk of remnant gastric cancer and opt for this procedure. To clarify potential heterogeneity, we conducted a subgroup analysis (Supplementary Table 9). Patients in the resectional group had longer diabetes duration, higher preoperative HbA1c, longer operative time, and longer hospital stay. At 1 year, BMI and %TWL were comparable between groups, but diabetes and dyslipidemia remission rates were lower in the resectional group, likely reflecting their more severe baseline diabetes rather than an intrinsic difference in procedure efficacy. Given the small number of resectional cases (*n* = 16), direct comparisons between the two subtypes should be interpreted with caution.

In conclusion, this study did not demonstrate a statistically significant difference in T2DM remission rates between SG and RYGB at 1 and 2 years after IPTW adjustment in Korean patients. Diabetes duration and insulin use emerged as stronger predictors of remission than procedure type. However, the limited sample size, considerable attrition, and baseline imbalance in T2DM severity mean that these findings should be interpreted with caution. Under comparable baseline conditions, RYGB may provide greater metabolic benefits, a hypothesis that requires confirmation in larger, stratified randomized controlled trials.

## Supplementary Information

Below is the link to the electronic supplementary material.

Supplementary Material 1 (DOCX 46.5 KB)

Supplementary Material 2 (PNG 727 KB)

## Data Availability

No datasets were generated or analysed during the current study.
