# Correction: Metabolic Surgery for Obese Type 2 Diabetes: Korean Multicenter Cohort Study

**DOI:** 10.1007/s11695-025-08413-3

**Published:** 2025-12-12

**Authors:** Young Suk Park, Soo Min Ahn, Sang Hyun Kim, Sung Il Choi, Kyung Won Seo, Han Hong Lee, Youngsung Suh, Ji Yeon Park, Sang Eok Lee, Sungsoo Park, Dong Jin Kim, In Cho, Yoo Min Kim, Songchang Shi, Tae Jung Oh, Yun-Suhk Suh, Ki Hyun Kim, Seungwan Ryu, Mi Kyung Kim, Do Joong Park, Seong-Ho Kong, Young Min Cho, In Gyu Kwon, Jong Suk Park, Minyoung Lee, Hyuk-Joon Lee

**Affiliations:** 1 Department of Surgery, Seoul National University Bundang Hospital, Seoul National University College of Medicine, Seongnam, Seoul, Korea, Republic of; 2 Department of Surgery, Gangnam Severance Hospital, Yonsei University College of Medicine, Seoul, Korea, Republic of; 3 Department of Surgery, Soonchunhyang University Hospital, Seoul, Korea, Republic of; 4 Department of Surgery, Kyung Hee University Hospital at Gangdong, Seoul, Korea, Republic of; 5 Department of Surgery, Kosin University College of Medicine, Busan, Korea, Republic of; 6 Department of Surgery, Seoul St. Mary’s Hospital, College of Medicine, The Catholic University of Korea, Seoul, Korea, Republic of; 7 Department of Family Medicine, Keimyung University School of Medicine, Daegu, Korea, Republic of; 8 Department of Surgery, School of Medicine, Kyungpook National University, Kyungpook National University Chilgok Hospital, Daegu, Korea, Republic of; 9 Department of General Surgery, Konyang University Hospital, Daejeon, Korea, Republic of; 10 Department of Surgery, Korea University College of Medicine, Seoul, Korea, Republic of; 11 Department of Gastrointestinal Surgery, Eunpyeong St. May’s Hospital, College of Medicine, The Catholic University of Korea, Seoul, Korea, Republic of; 12 Department of Surgery, Soonchunhyang University Bucheon Hospital, Soonchunhyang University College of Medicine, Bucheon, Korea, Republic of; 13 Department of Surgery, Republic of Korea Gastric Cancer Center, Yonsei Cancer Center, Yonsei University Health System, Yonsei University College of Medicine, Seoul, Korea, Republic of; 14 Department of Critical Care Medicine, Fujian Provincial Hospital, Fujian Provincial Hospital South Branch, Shengli Clinical Medical College of Fujian Medical University, Fuzhou University Affiliated Provincial Hospital, Fuzhou, China; 15 Department of Internal Medicine, Seoul National University College of Medicine and Seoul National University Bundang Hospital, Seongnam-si, Korea, Republic of; 16 Department of Surgery, Seoul National University Bundang Hospital, Seoul National University College of Medicine, Seongnam, Seoul, Korea, Republic of; 17 Department of Surgery, Keimyung University School of Medicine, Daegu, Korea, Republic of; 18 Department of Surgery and Cancer Research Institute, Seoul National University Hospital, Seoul National University College of Medicine, Seoul, Korea, Republic of; 19 Department of Internal Medicine, Seoul National University Hospital, Seoul National University College of Medicine, Seoul, Korea, Republic of; 20 Department of Internal Medicine, Gangnam Severance Hospital, Yonsei University College of Medicine, Seoul, Korea, Republic of; 21 Department of Internal Medicine, Yonsei University College of Medicine, Seoul, Korea, Republic of

**Correction: Obesity Surgery**

10.1007/s11695-025-08374-7

There is blinded information in the Acknowledgements. The correct version follows:

Acknowledgement: This research was supported by the National Evidence-based Healthcare Collaborating Agency (NECA) of Korea under the research grant number HC21C0099.

