# Supplementary material for: Metabolic Surgery for Obese Type 2 Diabetes: Korean Multicenter Cohort Study
Source: Obes Surg. 2025 Nov 13;35(12):5441–51. doi: 10.1007/s11695-025-08374-7 (PMC12722412; doi:10.1007/s11695-025-08374-7)
Supplement: Supplementary file 1 — Supplementary Material 1 (DOCX 46.5 KB) [file 11695_2025_8374_MOESM1_ESM.docx]

| Supplementary Table 1. Distribution of patients by center and procedure | | |  |
| --- | --- | --- | --- |
| Center | Total | SG | RYGB |
| Seoul National University Bundang Hospital | 94 | 84 | 10 |
| Soonchunhyang University Hospital | 50 | 32 | 18 |
| Kyung Hee University Hospital at Gangdong | 61 | 16 | 45 |
| Kosin University Hospital | 52 | 25 | 27 |
| Seoul St. Mary’s Hospital | 39 | 33 | 6 |
| Keimyung University Hospital | 13 | 12 | 1 |
| Seoul National University Hospital | 27 | 21 | 6 |
| Kyungpook National University Chilgok Hospital | 19 | 7 | 12 |
| Konyang University Hospital | 21 | 14 | 7 |
| Korea University Anam Hospital | 18 | 18 | 0 |
| Eunpyeong St. Mary's Hospital | 13 | 11 | 2 |
| Soonchunhyang University Bucheon Hospital | 13 | 9 | 4 |
| Gangnam Severance Hospital | 10 | 5 | 5 |
| Severance Hospital | 5 | 5 | 0 |

| Supplementary Table 2. Baseline characteristics of the 1-year follow-up cohort | | | |  |  |  |  |  |  |
| --- | --- | --- | --- | --- | --- | --- | --- | --- | --- |
|  | Before IPTW |  |  | |  | After IPTW |  |  |  |
|  | Sleeve gastrectomy | Roux-en-Y Gastric bypass | p | |  | Sleeve gastrectomy | Roux-en-Y Gastric bypass | p | SMD |
|  | (N = 166) | (N = 105) |  |  |  | (N = 308.5) | (N = 271.9) |  |  |
| Sex, female | 88 (53.0%) | 45 (42.9%) | 0.132 | |  | 125.1(40.6%) | 124.2(45.7%) | 0.635 | 0.103 |
| Age, yrs | 43.3 ± 12.8 | 46.9 ± 12.1 | 0.021 | |  | 48.3 ± 14.2 | 44.5 ± 12.7 | 0.296 | 0.068 |
| Smoking |  |  | 0.004 | |  |  |  | 0.743 | 0.160 |
| Never-smoker | 116 (69.9%) | 75 (71.4%) |  | |  | 238.5 (77.3%) | 195.3 (71.8%) |  |  |
| Current smoker | 34 (20.5%) | 9 (8.57%) |  | |  | 41.2 (13.3%) | 42.9 (15.8%) |  |  |
| Ex-smoker | 16 (9.64%) | 21 (20.0%) |  | |  | 28.8 (9.3%) | 33.7 (12.4%) |  |  |
| BMI, kg/m^2^ | 40.3 ± 6.64 | 37.7 ± 5.47 | 0.001 | |  | 38.5 ± 6.66 | 40.9 ± 7.35 | 0.215 | 0.117 |
| Hypertension | 121 (72.9%) | 81 (77.1%) | 0.522 | |  | 241 (78.1%) | 215.9 (79.4%) | 0.853 | 0.067 |
| Dyslipidemia | 107 (64.5%) | 83 (79.0%) | 0.016 | |  | 231.1 (74.9%) | 194.9 (71.7%) | 0.720 | 0.016 |
| Chronic heart disease | 14 (8.43%) | 18 (17.1%) | 0.049 | |  | 27.4 (8.9%) | 29.8 (11.0%) | 0.620 | 0.070 |
| NAFLD | 93 (56.0%) | 78 (74.3%) | 0.004 | |  | 212.1 (68.8%) | 194.2 (71.4%) | 0.773 | 0.184 |
| OSA | 73 (44.0%) | 60 (57.1%) | 0.047 | |  | 174.0 (56.4%) | 115.2 (42.4%) | 0.190 | 0.069 |
| Psychotic disease | 38 (22.9%) | 22 (21.0%) | 0.822 | |  | 60.0 (19.5%) | 70.7 (26.0%) | 0.424 | 0.054 |
| GERD, subjective | 46 (27.7%) | 30 (28.6%) | 0.988 | |  | 78.3 (25.4%) | 72.4 (26.6%) | 0.894 | 0.106 |
| DM duration, yrs | 3.14 ± 4.69 | 6.98 ± 7.45 | <0.001 | |  | 4.97 ± 5.86 | 4.71 ± 6.19 | 0.663 | 0.089 |
| Retinopathy |  |  | 0.106 | |  |  |  | 0.967 | 0.035 |
| Yes | 8 (4.82%) | 10 (9.52%) |  | |  | 16.1(5.2%) | 16.4(6.0%) |  |  |
| Unknown | 32 (19.3%) | 27 (25.7%) |  | |  | 61.4(19.9%) | 19.7(53.7%) |  |  |
| Nephropathy |  |  | 0.002 | |  |  |  | 0.297 | 0.337 |
| Yes | 6 (3.61%) | 16 (15.2%) |  | |  | 60.9(19.7%) | 21.9(8.0%) |  |  |
| Unknown | 29 (17.5%) | 20 (19.0%) |  | |  | 57(18.5%) | 45.5(16.7%) |  |  |
| Neuropathy |  |  | 0.265 | |  |  |  | 0.195 | 0.383 |
| Yes | 9 (5.42%) | 11 (10.5%) |  | |  | 62.7(20.3%) | 19.4(7.1%) |  |  |
| Unknown | 23 (13.9%) | 16 (15.2%) |  | |  | 47(15.2%) | 33.8(12.4%) |  |  |
| Insulin use | 9 (5.42%) | 11 (10.5%) | 0.265 | |  | 90.7(29.4%) | 34(15.8%) | 0.205 | 0.325 |
| C-peptide, ng/mL | 5.06 ± 3.59 | 4.15 ± 3.10 | 0.029 | |  | 4.59 ± 3.10 | 5.05 ± 4.00 | 0.567 | 0.008 |
| HbA1C, % | 7.04 (1.19) | 7.82 (1.47) | <0.001 | |  | 7.55 ± 1.40 | 7.30 ± 1.33 | 0.458 | 0.003 |
| BMI, body mass index; NAFLD, non-alcoholic fatty liver disease; OSA, obstructive sleep apnea; DM, diabetes mellitus; GERD, gastroesophageal reflux disease | | | | | | | | |  |

| Supplementary Table 3. Baseline characteristics of the 2-year follow-up cohort | | | |  |  |  |  |  |  |
| --- | --- | --- | --- | --- | --- | --- | --- | --- | --- |
|  | Before IPTW |  |  | |  | After IPTW |  |  |  |
|  | Sleeve gastrectomy | Roux-en-Y Gastric bypass | p | |  | Sleeve gastrectomy | Roux-en-Y Gastric bypass | p | SMD |
|  | (N = 105) | (N = 64) |  |  |  | (N = 196.5) | (N = 145.9) |  |  |
| Sex, female | 60 (57.1%) | 45 (70.3%) | 0.121 | |  | 138.3(70.4%) | 91.6(62.8%) | 0.548 | 0.163 |
| Age, yrs | 44.0 ± 13.0 | 46.3 ± 11.6 | 0.235 | |  | 49.1 ± 14.4 | 47.0 ± 11.2 | 0.644 | 0.158 |
| Smoking |  |  | <0.001 | |  |  |  | 0.560 | 0.177 |
| Never-smoker | 76 (72.4%) | 46 (71.9%) |  | |  | 157.6(80.2%) | 113.1(77.5%) |  |  |
| Current smoker | 23 (21.9%) | 3 (4.69%) |  | |  | 25.5(13.0%) | 13.4(9.2%) |  |  |
| Ex-smoker | 6 (5.71%) | 15 (23.4%) |  | |  | 13.3(6.8%) | 19.3(13.3%) |  |  |
| BMI, kg/m^2^ | 40.4 ± 7.34 | 37.6 ± 5.55 | 0.005 | |  | 37.7 ± 7.17 | 38.0 ± 5.36 | 0.856 | 0.041 |
| Hypertension | 76 (72.4%) | 49 (76.6%) | 0.674 | |  | 154.3(78.5%) | 101.7(69.7%) | 0.433 | 0.192 |
| Dyslipidemia | 67 (63.8%) | 48 (75.0%) | 0.179 | |  | 143.5(73.1%) | 110.4(75.7%） | 0.788 | 0.064 |
| Chronic heart disease | 10 (9.52%) | 12 (18.8%) | 0.135 | |  | 17.2(8.7%) | 25.3(17.3%) | 0.229 | 0.238 |
| NAFLD | 51 (48.6%) | 46 (71.9%) | 0.005 | |  | 130.1(66.2%) | 104.3(71.5%) | 0.653 | 0.143 |
| OSA | 43 (41.0%) | 33 (51.6%) | 0.236 | |  | 109.2(55.6%) | 55.7(38.2%) | 0.208 | 0.204 |
| Psychotic disease | 21 (20.0%) | 16 (25.0%) | 0.568 | |  | 34.2(17.4%) | 42.7(29.3%) | 0.260 | 0.259 |
| GERD, subjective | 34 (32.4%) | 15 (23.4%) | 0.285 | |  | 42.5(21.6%) | 35.7(24.5%) | 0.770 | 0.051 |
| DM duration, yrs | 4.77 ± 5.92 | 7.91 ± 8.28 | 0.004 | |  | 5.26 ± 5.38 | 6.03 ± 7.34 | 0.952 | 0.114 |
| Retinopathy |  |  | 0.313 | |  |  |  | 0.774 | 0.072 |
| Yes | 8 (7.62%) | 8 (12.5%) |  | |  | 11.7(5.9%) | 12.8(8.8%) |  |  |
| Unknown | 14 (13.3%) | 12 (18.8%) |  | |  | 23.2(11.8%) | 18.8(12.9%) |  |  |
| Nephropathy |  |  | 0.052 | |  |  |  | 0.257 | 0.391 |
| Yes | 5 (4.76%) | 10 (15.6%) |  | |  | 52.8(26.9%) | 14.9(10.2%) |  |  |
| Unknown | 13 (12.4%) | 8 (12.5%) |  | |  | 19.2(9.8%) | 13.6(9.3%) |  |  |
| Neuropathy |  |  | 0.004 | |  |  |  | 0.580 | 0.308 |
| Yes | 3 (2.86%) | 10 (15.6%) |  | |  | 50.7(25.8%) | 21.3(14.6%) |  |  |
| Unknown | 9 (8.57%) | 9 (14.1%) |  | |  | 15.1(7.7%) | 14.5(9.9%) |  |  |
| Insulin use | 11 (10.5%) | 20 (31.2%) | 0.001 | |  | 67.0(34.1%) | 28.9(19.8%) | 0.332 | 0.326 |
| C-peptide, ng/mL | 4.93 ± 3.54 | 4.43 ± 3.32 | 0.359 | |  | 4.57 ± 3.05 | 4.37 ± 2.93 | 0.692 | 0.073 |
| HbA1C, % | 7.05 ± 1.30 | 7.93 ± 1.40 | <0.001 | |  | 7.69 ± 1.51 | 7.33 ± 1.36 | 0.435 | 0.147 |
| BMI, body mass index; NAFLD, non-alcoholic fatty liver disease; OSA, obstructive sleep apnea; DM, diabetes mellitus; GERD, gastroesophageal reflux disease | | | | | | | | |  |

| Supplementary Table 4. Postoperative morbidity |  |  |  |
| --- | --- | --- | --- |
|  | Sleeve gastrectomy | Roux-en-Y Gastric bypass | *p* |
|  | (N=292) | (N=143) |  |
| Major early complications (%) | 11 (3.8) | 10 (7.0) | 0.216 |
| Major late complications (%) | 2 (0.7) | 2 (1.4) | 0.843 |
| Anastomosis leakage (%) | 2 (0.7) | 0 | 0.812 |
| Bleeding (%) | 5 (1.7) | 2 (1.4) | 0.999 |
| Bowel perforation (%) | 0 | 0 | 0.999 |
| SB obstruction (%) | 0 | 0 | 0.999 |
| Trocar site hernia (%) | 0 | 2 (1.4) | 0.204 |
| Renal failure (%) | 0 | 0 | 0.999 |
| Respiratory failure (%) | 0 | 0 | 0.999 |
| Prolonged hospital stay (%) | 8 (2.7) | 10 (7.0) | 0.066 |
| Major nausea and vomiting (%) | 0 | 0 | 0.999 |
| Sleeve stenosis (%) | 0 | 0 | 0.999 |
| Major SSI (%) | 1 (0.3) | 0 | 0.999 |
| VTE (%) | 1 (0.3) | 0 | 0.999 |
| Minor early complications (%) | 17 (5.8) | 2 (1.4) | 0.061 |
| Minor late complications (%) | 7 (2.4) | 7 (4.9) | 0.272 |
| Marginal ulcer (%) | 0 | 3 (2.1) | 0.062 |
| Anastomosis stricture (%) | 2 (0.7) | 0 | 0.812 |
| Minor nausea and vomiting (%) | 6 (2.1) | 1 (0.7) | 0.516 |
| ARF (%) | 0 | 1 (0.7) | 0.715 |
| Ileus (%) | 5 (1.7) | 2 (1.4) | 0.999 |
| Minor SSI (%) | 2 (0.7) | 1 (0.7) | 0.999 |
| Dehydration (%) | 2 (0.7) | 2 (1.4) | 0.843 |
| UTI (%) | 0 | 0 | 0.999 |
| Symptomatic GB stones (%) | 0 | 0 | 0.999 |
| SB, small bowel; SSI, surgical site infection; VTE, venous thromboembolism; ARF, acute renal failure; UTI, urinary tract infection; GB, gall bladder | | | |

| Supplementay Table 5. Predictors of diabetes remission (ordinary logistic regression vs. mixed-effects logistic regression with random intercept for center) | | |
| --- | --- | --- |
| A. Postoperative 1-year |  |  |
| Variable | Multivariable OR (95% CI), p | Mixed-effects OR (95% CI), p |
| Age | 0.99 (0.96–1.03), p=0.717 | 0.99 (0.95–1.03), p=0.642 |
| RYGB vs SG | 1.63 (0.74–3.79), p=0.239 | 1.52 (0.69–3.54), p=0.298 |
| BMI | 1.05 (0.98–1.13), p=0.150 | 1.04 (0.97–1.12), p=0.212 |
| DM duration | 0.76 (0.68–0.84), p<0.001 | 0.77 (0.69–0.85), p<0.001 |
| Insulin use | 0.29 (0.10–0.84), p=0.023 | 0.31 (0.11–0.90), p=0.032 |
| C-peptide | 0.99 (0.88–1.13), p=0.882 | 1.01 (0.89–1.15), p=0.861 |
| AST | 1.00 (0.98–1.02), p=0.954 | 1.00 (0.98–1.02), p=0.944 |
| ALT | 1.00 (0.98–1.01), p=0.856 | 1.00 (0.98–1.01), p=0.872 |
|  |  |  |
| B. Postoperative 2-year |  |  |
| Variable | Multivariable OR (95% CI), p | Mixed-effects OR (95% CI), p |
| Age | 0.98 (0.93–1.03), p=0.438 | 0.98 (0.93–1.04), p=0.464 |
| RYGB vs SG | 0.76 (0.29–2.04), p=0.576 | 0.79 (0.30–2.14), p=0.640 |
| BMI | 1.13 (1.02–1.28), p=0.044 | 1.11 (0.99–1.26), p=0.067 |
| DM duration | 0.86 (0.77–0.94), p=0.002 | 0.87 (0.78–0.95), p=0.004 |
| Insulin use | 0.25 (0.06–0.86), p=0.033 | 0.27 (0.07–0.91), p=0.038 |
| C-peptide | 1.10 (0.91–1.37), p=0.333 | 1.12 (0.92–1.40), p=0.308 |
| AST | 1.00 (0.97–1.04), p=0.835 | 1.00 (0.97–1.04), p=0.842 |
| ALT | 1.01 (0.99–1.04), p=0.495 | 1.01 (0.99–1.04), p=0.488 |

| Supplementary Table 6. Remission by IMS stratification | | |  |  |  |
| --- | --- | --- | --- | --- | --- |
| IMS group | SG | Remission (Complete+Partial) | RYGB | Remission (Complete+Partial) | p-value |
| Mild (0-25) | 61 | 56 (91.8%) | 19 | 18 (94.7%) | 0.999 |
| Moderate (26-95) | 93 | 67 (72.0%) | 59 | 39 (66.1%) | 0.472 |
| Severe (96) | 12 | 2 (16.7%) | 27 | 4 (14.8%) | 0.999 |

| Supplementray Table 7. Multivariable logistric regression analysis including IMS | | | |  |
| --- | --- | --- | --- | --- |
|  | OR | 95% CI lower | 95% CI upper | p-value |
| Age (continuous) | 0.973 | 0.935 | 1.011 | 0.165 |
| BMI (continuous) | 1.061 | 0.988 | 1.139 | 0.105 |
| DM duration (continuous) | 0.915 | 0.795 | 1.053 | 0.217 |
| Insulin use | 0.556 | 0.183 | 1.692 | 0.301 |
| C-peptide (continuous) | 0.979 | 0.848 | 1.131 | 0.777 |
| HbA1c (continuous) | 0.774 | 0.55 | 1.09 | 0.142 |
| AST (continuous) | 1.01 | 0.978 | 1.023 | 0.967 |
| ALT (continuous) | 1.02 | 0.984 | 1.016 | 0.97 |
| Operative procedure (Roux-en-Y gastric bypass) | 2.058 | 0.856 | 4.953 | 0.107 |
| IMS (continuous) | 0.969 | 0.945 | 0.993 | 0.011 |

| Supplementary Table 8. Baseline Characteristics of Patients by Follow-up Status at 1 and 2 Years | | |  |
| --- | --- | --- | --- |
| Variable | Completers at 1 year (N=271) | Lost to follow-up (N=164) | p-value |
| Age, yrs (mean ± SD) | 44.6 ± 12.1 | 42.8 ± 13.0 | 0.18 |
| Female sex, n (%) | 133 (49.1%) | 145 (88.4%) | <0.001 |
| Baseline BMI, kg/m² | 39.5 ± 6.3 | 40.2 ± 6.6 | 0.29 |
| Diabetes duration, yrs | 4.7 ± 6.0 | 4.1 ± 6.3 | 0.42 |
| Insulin use, n (%) | 58 (21.4%) | 42 (25.6%) | 0.31 |
| Any comorbidity*, n (%) | 226 (83.3%) | 131 (79.9%) | 0.37 |
|  |  |  |  |
| Variable | Completers at 2 years (N=169) | Lost to follow-up (N=266) | p-value |
| Age, yrs (mean ± SD) | 45.2 ± 12.3 | 43.1 ± 12.9 | 0.12 |
| Female sex, n (%) | 105 (62.1%) | 173 (65.0%) | 0.55 |
| Baseline BMI, kg/m² | 39.0 ± 6.4 | 39.8 ± 6.2 | 0.28 |
| Diabetes duration, yrs | 5.8 ± 7.2 | 4.2 ± 6.1 | 0.03 |
| Insulin use, n (%) | 31 (18.3%) | 62 (23.3%) | 0.23 |
| Any comorbidity*, n (%) | 142 (84.0%) | 218 (82.0%) | 0.61 |
| *Any comorbidity was defined as the presence of at least one obesity-related comorbidity (hypertension, dyslipidemia, NAFLD, OSA, etc.) other than diabetes. | | | |

| Supplementay Table 9. Comparison of conventional vs. resectional RYGB at baseline, operative, and 1-year outcomes | | | |
| --- | --- | --- | --- |
| Variable | Conventional RYGB (n=89) | Resectional RYGB (n=16) | p-value |
| Baseline characteristics |  |  |  |
| Male sex (%) | 69.70% | 81.30% | 0.4 |
| Age (years) | 47.0 ± 12.2 | 46.3 ± 11.9 | 0.83 |
| BMI (kg/m²) | 37.8 ± 5.5 | 37.0 ± 5.6 | 0.56 |
| DM duration (years) | 6.6 ± 7.8 | 8.7 ± 5.7 | 0.042 |
| HbA1c (%) | 7.7 ± 1.4 | 8.5 ± 1.5 | 0.036 |
| C-peptide (ng/mL) | 4.1 ± 2.8 | 3.4 ± 2.1 | 0.32 |
| Operative outcomes |  |  |  |
| Operation time (min) | 168.3 ± 72.3 | 183.5 ± 36.1 | 0.048 |
| Hospital stay (days) | 5.6 ± 2.2 | 8.1 ± 2.2 | <0.001 |
| Major early complication (%) | 2.20% | 6.30% | 0.36 |
| Major late complication (%) | 2.20% | 0% | 0.53 |
| Readmission (%) | 4.50% | 12.50% | 0.27 |
| Reoperation (%) | 2.20% | 0% | 0.53 |
| Weight loss outcomes |  |  |  |
| Weight (kg) | 78.1 ± 15.0 | 74.2 ± 15.2 | 0.24 |
| BMI (kg/m²) | 28.5 ± 3.6 | 27.8 ± 4.7 | 0.47 |
| %TWL | 24.5 ± 7.4% | 24.9 ± 8.6% | 0.77 |
| HTN remission |  |  | 0.174 |
| Complete remission | 15 (16.9%) | 4 (25.0%) |  |
| Partial remission | 17 (19.1%) | 0 |  |
| Improvement | 22 (24.7%) | 5 (31.3%) |  |
| No change | 3 (3.4%) | 0 |  |
| Progression | 1 (1.1%) | 1 (6.3%) |  |
| Dyslipidemia remission |  |  | 0.044 |
| Remission | 36 (40.4%) | 1 (6.3%) |  |
| Improvement | 28 (31.5%) | 6 (37.5%) |  |
| No change | 3 (3.4%) | 2 (12.5%) |  |
| Progression | 1 (1.1%) | 0 |  |
| DM remission |  |  | <0.001 |
| Complete remission | 50 (56.2%) | 4 (25.0%) |  |
| Partial remission | 7 (7.9%) | 0 |  |
| Improvement | 31 (34.8%) | 9 (56.3%) |  |
| No change | 1 (1.1%) | 3 (18.8%) |  |
