# Supplementary figures and images for: Metabolic Surgery for Obese Type 2 Diabetes: Korean Multicenter Cohort Study
Source: Obes Surg. 2025 Nov 13;35(12):5441–51. doi: 10.1007/s11695-025-08374-7 (PMC12722412; doi:10.1007/s11695-025-08374-7)

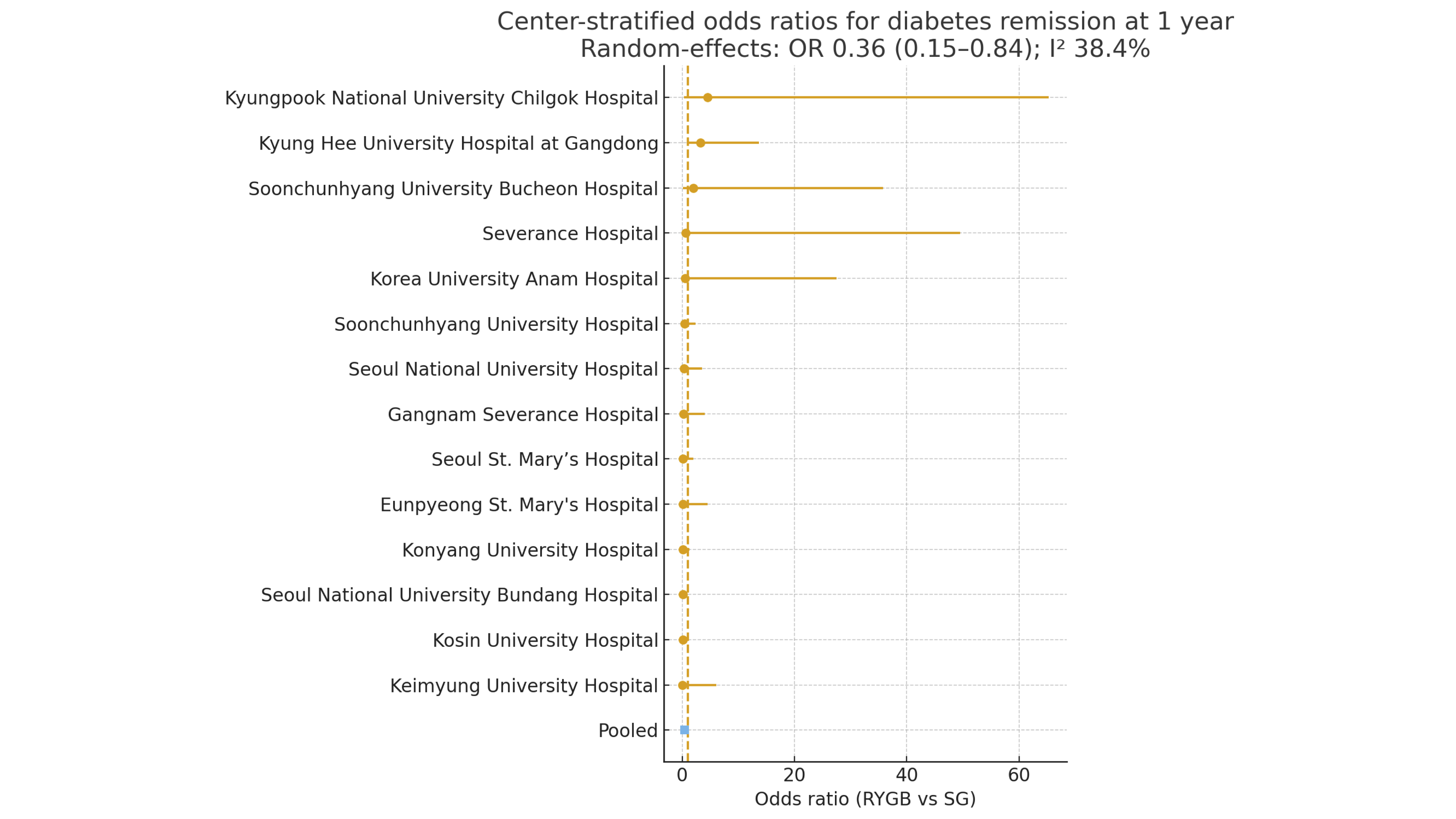

Supplement: Supplementary file 2 — Supplementary Material 2 (PNG 727 KB) [file 11695_2025_8374_MOESM2_ESM.png]
